# Evaluating concurrent validity of criminal justice and clinical assessments among women on probation

**DOI:** 10.1186/s40352-018-0065-6

**Published:** 2018-04-07

**Authors:** Camila D. Odio, Megan Carroll, Susan Glass, Ashley Bauman, Faye S. Taxman, Jaimie P. Meyer

**Affiliations:** 1 Department of Internal Medicine, Yale New Haven Health, PO Box 208030, New Haven, CT 06520-8030 USA; 2 Department of Biostatistics, Yale School of Public Health, New Haven, CT USA; 3 Department of Epidemiology, Johns Hopkins Bloomberg School of Public Health, 615 N. Wolfe Street, Baltimore, MD 21205 USA; 4 Connecticut Judicial Branch, Court Support Services Division, 936 Silas Deane Hwy, Wethersfield, CT 06109 USA; 5 Bauman Consulting Group, LLC, 411 W. Loveland Ave., Suite 201-B, Loveland, OH 45140 USA; 6 Criminology, Law & Society, George Mason University, 4400 University Drive, 4F4, Fairfax, VA 22030 USA; 7 AIDS Program, Yale School of Medicine, 135 College Street, Suite 323, New Haven, CT 06510 USA

**Keywords:** Women, Probation, Criminal justice, HIV, Risk needs assessment

## Abstract

**Background:**

Women in the criminal justice (CJ) system experience complex and comorbid medical, psychiatric, and substance use disorders, which often contribute to CJ involvement. To identify intersections between CJ and health needs, we calculated Spearman r correlations between concurrent CJ and clinical assessments from women on probation in Connecticut who were enrolled in a clinical trial. We examined longitudinal trends in CJ risk scores over 9 years of observation (2005–2014), modeling time to probation recidivism with shared gamma frailty models and comparing contiguous time points by Wilcoxon matched-pairs signed rank tests.

**Results:**

Women (*N* = 31) were predominantly white (67.7%) with at least some high school education (58.1%) and mostly unemployed (77.4%) and unstably housed (83.9%). Most met clinical criteria for severe substance use and/or psychiatric disorders. Concurrent measures of substance use, mental health, social support, partnerships, and risk by the Level of Service Inventory-Revised (LSI-R) and clinical assessments were not significantly correlated. The LSI-R personal/emotional sub-score, however, positively correlated with the Addiction Severity Index psychiatric composite score (*r* = 0.40, 95% CI 0.03–0.68, *p* = 0.03). After adjusting for age, race and number of previous events, having some high school education versus none marginally decreased the hazard for probation recidivism and having > 5 inpatient psychiatric admissions versus none increased the hazard of probation recidivism 7-fold (HR 7.49, 95% CI 1.33–42.12, *p =* 0.022). Women with 0–1 recurrent probation terms (*n* = 16) had a significantly lower mean LSI-R score than those with 2–4 recurrent probation terms (35.9 [SD 6.4] versus 39.2 [SD 3.0], *p* = 0.019), but repeated LSI-R scores did not change over time, nor vary significantly beyond the group mean.

**Conclusions:**

In this small, quantitative study of women on probation, widely used CJ assessment tools poorly reflected women’s comorbid medical, psychiatric, and substance use needs and varied minimally over time. Findings illustrate the limitations of contemporary CJ assessment tools for women with complex needs. The field requires more comprehensive assessments of women’s social and health needs to develop individualized targeted case plans that simultaneously improve health and CJ outcomes.

## Background

The US incarcerates more of its citizens, and more women, than any other nation worldwide (Pew Chartitable Trusts, 2009). At year-end 2015, 2.7% of all US adults were under some form of correctional supervision, including prison, jail, probation and parole, and 1.9% were on probation or parole (Kaeble, 2016). Compared to the general adult population, people involved in criminal justice (CJ) systems disproportionately experience co-occurring medical, psychiatric, and substance use disorders (Bronson et al., 2017), and report four times more adverse childhood events (Reavis et al., 2013). More than half of all inmates meet criteria for substance abuse, and two-thirds of female and one-third of male prisoners have a diagnosed psychiatric disorder (Bronson & Berzofsky, 2017). Compared to CJ-involved men, CJ-involved women have a higher burden of complex comorbidities, including HIV (Binswanger et al., 2010). Women involved in CJ systems thus experience a set of unique vulnerabilities.

Incarceration is destabilizing and has detrimental effects on health (Binswanger et al., 2012). CJ involvement often disrupts continuity of care for chronic health conditions, including HIV, and contributes to poor health outcomes when resources are not provided during or following imprisonment (Meyer et al., 2014; Rich et al., 2015). Women are particularly vulnerable to disrupted care because they are more likely than their male counterparts to have experienced violence victimization and have untreated psychiatric and substance use disorders, including depression and post-traumatic stress (Women in the Criminal Justice System Briefing Sheets, 2007). Some women report substance use as a coping mechanism for traumatic life events (Morash, 2010), and fewer than 20% receive substance abuse treatment and 25% receive mental health services while involved with the criminal justice system (McCampbell, 2005).

Unrecognized or untreated psychiatric and substance use disorders are associated with recidivism and poor CJ outcomes (Fu et al., 2013; Baillargeon et al., 2010). Conversely, recognition and treatment of health needs, particularly substance use disorders, have been linked to decreased recidivism (Wooditch et al., 2014; Lamberti, 2016). In addition, lower rates of recidivism are reported among those who are employed (Holzer & Raphael, 2018), but poor health is a risk factor for unemployment (van Rijn et al., 2014). Women’s health has even broader reaching effects, as more than half of CJ-involved women are mothers (Glaze & Maruschak, 2010), and poor maternal health is associated with behavioral problems, poor health outcomes, and delayed verbal development in their children (Hardie & Turney, 2017).

As women interface with health and CJ systems, issues can potentially be identified so that they may be linked to care and services. Yet these systems are often difficult to navigate, particularly for women with limited health literacy or self-efficacy, and highly siloed. The seeming alignment between public health and public safety risks and outcomes place a burden on assessment protocols to reflect both CJ risk and need factors, including health issues that complicate outcomes. This raises issues related to the adequacy of the contemporary CJ risk and need assessments (RNA) to identify complex health issues. The field has developed an array of standardized instruments to measure static risk factors and dynamic needs and thereby efficiently triage large numbers of people in an era of mass incarceration (Monahan & Skeem, 2013). Criminal justice RNA are distinct from clinical assessments because they are focused on actuarial, rather than individual, factors that contribute to recidivism and they include a set of dynamic factors that are potentially modifiable (Taxman et al., 2006). In some states, RNA are used in criminal sanctioning; in others, scores are applied in post-judicial settings to develop and monitor case plans and to manage supervising officers’ caseloads (Kehl & Kessler, 2017). The most commonly used RNA instrument is the Level of Service Inventory-Revised (Lowenkamp et al., 2009), which has overall and sub-scores that are reliable and valid predictors of recidivism (Flores et al., 2006). Although there is limited data on how measured CJ risk factors and health risks interact, one study reported decreased longitudinal survival for individuals with high, compared to low, CJ risk (Folsom & Atkinson, 2007). Another cohort study of nearly 1000 adolescents demonstrated that co-occurring substance use and psychiatric disorders moderated the relationship between CJ risk markers and re-arrest (Schubert et al., 2011).

Because health and social factors are dynamic, additional concerns have been raised regarding the sensitivity of RNA instruments to adequately detect changes in individual-level factors over time (Wooditch et al., 2014; Schlager & Pacheco, 2011). Many of the dynamic needs are single item questions while others are questionnaires that include lifetime, last year and/or last month behavior. The dynamic need factors are often poorly conceived, lack content validity, and vary in measurement of concepts, limiting the utility of the RNA instrument to measure needs or to change over time (Via & Dezember, 2016).

Our small, quantitative study explores the intersections of health and CJ risk factors to better understand the dynamics of RNA measurements over time. We were particularly interested in how measurements of CJ risk reflect health risk for women. This is a secondary data analysis involving 31 women on probation, who underwent concurrent measurements of health and CJ risks as part of a clinical trial and probation supervision, allowing us to examine concurrent validity along several key domains. By reevaluating how CJ risk is measured, we hoped to identify modifiable factors that could affect women’s health and CJ outcomes, thus streamlining services for women and promoting women’s overall health.

## Methods

### Study setting

The study took place in Connecticut, wherein the state’s Judicial Branch, Court Support Services Division (CSSD), oversees adult probation for approximately 9000 women. For the approximately 750 women state-wide who are classified as “high risk” for re-offending, probation services are provided by the Women Offender’s Case Management program (WOCM), which includes gender-responsive case managers, specially trained probation officers, and women-specific “alternatives in the community” programming. HIV-related or other health-focused programming is not currently routinely incorporated into probation case plans.

### Study participants and procedures

The parent study, known as +Pink (registered as NCT #03175094 at clinicaltrials.gov) and described elsewhere (Marcus et al., 2017), was designed to inform, adapt, and pilot test a behavioral HIV risk reduction intervention for CJ-involved women. Women were referred to +Pink by trained study staff on-site at probation offices, and HIV and primary care clinics in New Haven and Hartford, Connecticut. They could also be referred to the study by probation officers, case managers, or community healthcare providers, or could self-refer using a Qualtrics link or leaving a message on a secured dedicated voicemail. Referred participants were screened by a trained research assistant for the following inclusion criteria: 1) age ≥ 18 years old; 2) identify as female (cis- or trans-gendered); 3) CJ-involved, meaning they were sentenced to probation or receiving intensive pretrial supervision by a probation officer, sentenced to parole, or released from jail or prison within 60 days; and 4) living with diagnosed HIV or at-risk for HIV. HIV risk was defined as ever having injected drugs, ever exchanging sex for money or goods, being diagnosed with a sexually transmitted infection (STI) within 90 days of enrollment, having unprotected sex with a partner whose HIV status is unknown or HIV+ in the 90 days prior to enrollment, or being incarcerated in prison or jail during the two years prior to enrollment. Probation status was preliminarily confirmed by searching the Judicial Branch website for publicly available records. Women were excluded if they were unable or unwilling to provide written informed consent, unable to comfortably converse in English, or were threatening to study staff. After providing written informed consent and officially enrolling in the study, participants were asked to sign a Release of Information, which included data from CSSD and Adult Probation.

### Data sources

For each study participant who allowed release of information from CSSD and Adult Probation, the unique probation identification number was confirmed using the individual’s first and last name, date of birth, and social security number. Date of study enrollment was matched to current probation term start and end dates to confirm probation status (i.e., currently sentenced to probation or receiving intensive pretrial supervision by a probation officer.) All women who self-reported being on probation had this status confirmed with a 100% match rate. Using the unique probation identification number, CSSD data managers then extracted all available demographic and CJ risk information collected between 2005 and 2016. As part of routine supervision procedures, within six months of entrance and ideally again during, and at the conclusion of each probation term, probation officers administer the Level of Service Inventory-Revised (LSI-R) in paper form (Bonta, 1995). Interviews are conducted in English or Spanish (with a phone-based translator), on a one-to-one basis with the probation officer and the participant. The instrument is divided into 10 subscales that include both dynamic and historical factors, each with a specific focus area of self-report, including: criminal history, education/employment, financial status, family/marital, accommodation (housing), leisure/recreation, companions, alcohol/drug problems, emotional/personal, and attitudes/orientation. Within each subscale, 2–10 yes or no questions are asked, for a total of 54 questions in the entire survey. Scores are totaled and categorized into low (0–21), medium (22–28), and high (> 29) risk groups (Ostermann & Herrschaft, 2013). The LSI-R is examined by CSSD on an individual case basis but has never been examined longitudinally in aggregate nor compared to health indicators. Probation officers also administer the Adult Substance Use Survey-Revised (ASUS-R) on intake (Alcohol and Drug Abuse Assessments, Surveys, and Software Programs, 2017). It is possible that probation officers independently collected additional information on women’s motivations for their behaviors, but this data is not routinely recorded in the central database. Extracted data was uploaded into password protected files and securely transferred to the research team for further analysis.

Clinical assessments were collected for each participant after enrollment in the clinical trial, as part of a baseline interview. Trained study staff conducted study interviews in English in a private setting using a structured protocol and validated instruments to measure substance use and substance use disorder severity, mental health, social support, partnerships, and HIV-related risk behaviors, as described further below. There was no qualitative data collected as part of the baseline study assessment. All data were entered into RedCAP. Clinical assessments were then merged with CSSD data using probation and study unique identifications numbers and securely shared among approved study team members for further analysis.

**Fig. 1:**
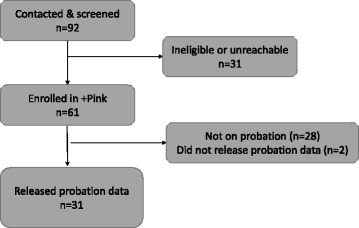
Flow of participants in +Pink Clinical Trial

As shown in Fig. 1, 92 women on probation were contacted and screened for eligibility for enrollment in the clinical trial, of whom 31 were either ineligible based on the pre-set inclusion/exclusion criteria (as described above) or were not reachable within the catchment area. Of the 61 women who were enrolled in the clinical trial, 28 were not on probation (i.e. they were on parole or were recently released from prison or jail) and 2 were on probation but did not consent to CSSD data release. Ultimately, 31 women released CSSD data (representing 108 assessments over 9 years of observation) and were included in the present analysis.

### Measures

Demographic data were obtained from CSSD records and included all available probation term start dates, age, race (categorized as black, white, or other), highest level of completed education (categorized as no, some, or completed high school), employment status (categorized as student, employed, unemployed, or disabled), and satisfaction with current housing accommodation.

*Substance use* was measured in the clinical trial by the Addiction Severity Index (ASI), 5th Edition alcohol and drug composite scores (McLellan et al., 1985). The ASI measures both past 30-day and lifetime substance use. Alcohol composite scores ≥0.17 indicate severe alcohol use disorders by DSM-IV criteria and drug composite scores ≥0.12 indicate severe substance use disorders by DSM-IV criteria (Rikoon et al., 2006). Substance use was measured by CSSD using the Level of Service Inventory-Revised (LSI-R (Bonta, 1995) alcohol/drug sub-scale and the ASUS-R, which asks about the number of times each of 9 substances have been used in one’s lifetime and in the past 6 months. The ASUS-R provides categorical levels of use for each substance (1 = one to 10 times; 2 = 11 to 25 times; 3 = 26 to 50 times; 4= > 50 times), giving a maximum score of 36 (Alcohol and Drug Abuse Assessments, Surveys, and Software Programs, 2017).

*Mental health* was measured in the clinical trial by the ASI psychiatric composite score, in which scores ≥0.22 are consistent with DSM-IV criteria for a severe psychiatric disorder (Calsyn et al., 2004), and the Quick Inventory of Depressive Symptomatology score (QIDS) (Rush et al., 2006). The QIDS is measured continuously with scores ranging 0–27 and higher scores indicate a more severe mood disorder; scores > 11 indicate major depression (Rush et al., 2003). Mental health was measured by CSSD using the LSI-R emotional/personal sub-scale.

*Social support* was measured in the clinical trial using the Social Support scale (Huba & Melchior, 1996), which is scored continuously from 0 to 100 with higher scores indicating greater social support; and in CSSD using the LSI-R family/marital and companions sub-scales.

*Partnerships and relationships* were measured in the clinical trial using the Revised Conflicts Tactics Scale-2 (STRAUS et al., 1996; STRAUS et al., 2003), which measures physical, sexual, emotional, and verbal intimate partner violence exposure and perpetration; and in CSSD with the LSI-R family/marital sub-scale.

*Risk* was measured in terms of HIV-related risk in the clinical trial using the NIDA Risk Behavior Assessment (Dowling-Guyer et al., 1994); and in CSSD in terms of criminogenic risk using the total LSI-R score. LSI-R results approaching the maximum score of 54 suggest higher criminogenic risk of “reoffending.”

### Statistical analysis

The concurrent validity of CSSD and clinical assessments within each domain described above (substance use, mental health, social support, relationships/partnerships, and risk) were estimated by Spearman r correlations, with *p <* 0.05 indicating statistical significance. For participants with multiple available CSSD assessments over the observation period, the most recent assessment was used, which was contemporaneous with clinical assessments. Trends of all LSI-R scores over time were visually examined and quantified by Wilcoxon matched-pairs signed rank tests comparing initial, reassessment and discharge LSI-R scores. These analyses were performed with PRISM version 7 for Mac OS X. Risk factors for time to recidivism to probation, defined by any new probation intake after completion of an initial term, were examined using a shared gamma frailty model adjusted for age, race and number of previous events. The shared gamma frailty model includes a random effect to account for the intra-subject correlation observed in repeated measures data. The probability of not having a recurrent probation intake over time was assessed by examining Kaplan-Meier curves. These analyses were performed using R version 3.3.2 and the “survival” package version 2.38.

## Results

As shown in Table 1, of the 31 women included in this secondary data analysis, the majority (58.1%) had some high school education, were unemployed (77.4%) and white (67.7%), and nearly all (83.9%) reported that their housing situation was inadequate, which essentially reflects the demographic profile of women on probation in the state. Participants’ mean LSI-R total score was 30.2 (SD 5.3), representing the highest CJ risk category. On further examination of potential contributing factors to CJ risk, many women identified current problems with alcohol (90.3%) or drugs (54.8%), and were receiving treatment for mental health issues, including multiple prior inpatient psychiatric admissions. Sixty-five percent of the women had a prior record of assault or violence. Three of the 31 participants were diagnosed and living with HIV, though many experienced significant HIV risk in terms of prior injection drug use (41.9%), transactional sex (32.3%), and sex with a partner with HIV or unknown sero-status (45.2%).

**Table 1:** Characteristics of Study Participants (*N* = 31)

Characteristics	N (%) or mean (SD)
Age, mean (SD)	33.7 (10.8)
Race, n (%)	
Black or Other	10 (32.3)
White	21 (67.7)
Education, n (%)	
No High School	13 (41.9)
Some High School	4 (12.9)
High School Graduate	14 (45.2)
Employment Status, n (%)	
Student/Employed	5 (12.1)
Unemployed	24 (77.4)
Disabled	2 (6.5)
Unsatisfactory Housing Accommodation, n (%)	26 (83.9)
Total LSI* Score, mean (SD)	30.23 (5.33)
Record of Assault/Violence, n (%)	20 (64.5)
Current Alcohol Problem, n (%)	28 (90.3)
Current Drug Problem, n (%)	17 (54.8)
Current Mental Health Problem, n (%)	13 (41.9)
History of Inpatient Treatment, n (%)	
Never	14 (45.2)
1–2 Times	11 (35.5)
3–5 Times	4 (12.9)
More than 5 times	2 (6.5)
HIV and Risk Factors, n (%)	
Diagnosed with HIV	3 (9.7)
Ever used injection drugs	13 (41.9)
Ever traded sex for drugs or money	10 (32.3)
Had unprotected sex with HIV+ or HIV status unknown partner in the past 90 days	14 (45.2)

*LSI level of service inventory

Of the 8 individuals with non-zero ASI alcohol composite scores, the mean was 0.17 (SD 0.16), which meets DSM-IV criteria for a severe alcohol use disorder. Of the 23 individuals with non-zero ASI drug composite scores, the mean was 0.11 (SD 0.10), which reflects severe substance use disorders. Of the 22 individuals with completed ASI psychiatric composite scores, the mean was 0.43 (SD 0.24), which is nearly double the cut-off criteria for severe psychiatric disorders; 8 women met clinical criteria for major depression by the QIDS.

Concurrent validity analyses revealed very few correlations between the CSSD surveys and the clinical assessments performed in four domains (Table 2). In the substance use domain, lifetime ASUS-R alcohol and drug scores were positively associated with the ASI, when only ASI items that quantified years of prior alcohol or drug use were included. Of the six LSI-R sub-score associations examined, only the LSI-R personal/emotional sub-score was positively correlated with a clinical assessment, the ASI psychiatric composite score. There was also no significant difference in median LSI-R family/marital sub-scores between women who did (*n* = 10) and did not (*n* = 14) report experiencing intimate partner violence in the past year on the Revised Conflicts Tactics Scale. When comparing overall CJ risk to HIV-related sexual risk behaviors, the correlation approached statistical significance when one high outlier was removed (*r* = 0.34, 95% CI: -0.06 to 0.64, *p =* 0.08).

**Table 2:**
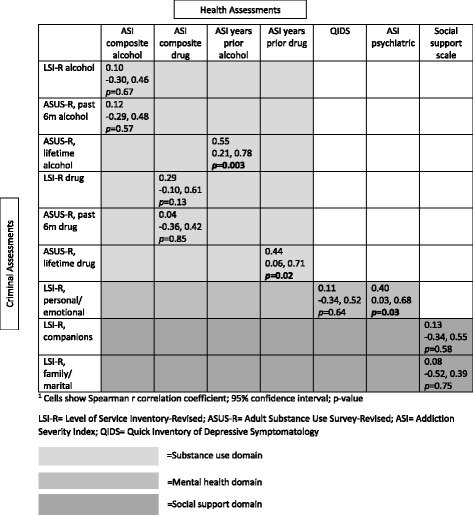
Correlations between criminal and health assessments, by domain (*N* = 31)

**Fig. 2:**
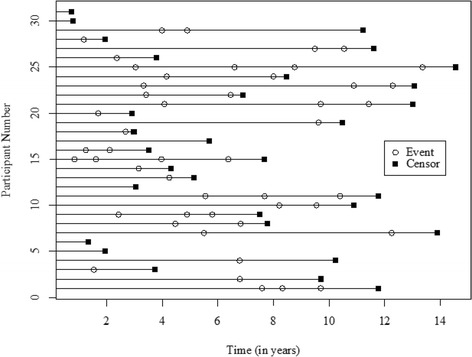
Individual trajectories over time with Risk and Need Assessments (*N* = 31)

Longitudinal examination of the 108 RNA assessments (including 80 intake assessments) revealed that six women had no further probation intakes after completing their first term, ten had one additional term and two had four additional terms (Fig. 2). Median time to recidivism for those with no prior probation terms was four years, but for those with one or two prior probation terms, the median time to recidivism decreased to three and two years respectively. The number of prior probation terms was inversely correlated with the median time to recidivism.

Table 3 shows the shared gamma frailty model of time to recidivism to probation. After adjusting for age, race and number of previous events, those with some high school education had a 60% lower hazard of reentry as compared to those with no high school education (HR: 0.40, 95% CI: 0.16 to 0.99, *p* = 0.048). Those with more than 5 inpatient psychiatric admissions had a 7.5 times greater hazard of recidivism as compared to those with less than five inpatient admissions (HR 7.49, 94% CI: 1.33 to 42.12, *p =* 0.02), though there were only two individuals who had > 5 inpatient admissions for psychiatric issues. Although the adjusted model did not reveal an association between total LSI scores and time to probation recidivism, women with 0–1 probation reentries (*n* = 16) did have significantly lower mean LSI-R scores than those with 2–4 reentries (35.9 [SD 6.4] versus 39.2 [SD 3.0], *p* = 0.019). Data was not available on whether participants were incarcerated in prison or jail during the observation period.

**Table 3:** Time to Recidivism by Shared Gamma Frailty Model (*N* = 31 women, 80 observations)

	HR (95% CI)^a^	*p*-value
Education		
No High School	1.00 (referent)	–
Some High School	*0.404 (0.16, 0.99)*	*0.048*
High School Graduate	0.752 (0.35, 1.61)	0.460
Unsatisfactory housing	1.577 (0.91, 4.08)	0.350
Total LSI score	0.951 (0.89, 1.02)	0.130
Record of assault/violence	*0.262 (0.09, 0.75)*	*0.013*
Current alcohol problem	0.871 (0.31, 2.42)	0.790
Current drug problem	1.059 (0.58, 1.94)	0.850
Current mental health problem	0.957 (0.53, 1.73)	0.890
History of inpatient psychiatric treatment		
Never Treated	1.00 (referent)	–
1–2 Times	1.429 (0.51, 4.01)	0.500
3–4 Times	2.09 (0.62, 7.10)	0.230
5 or more times	*7.49 (1.33, 42.12)*	*0.022*

*HR* hazard ratio, *LSI* level of service inventory

^a^adjusted for age, race, and number of previous events

**Fig. 3:**
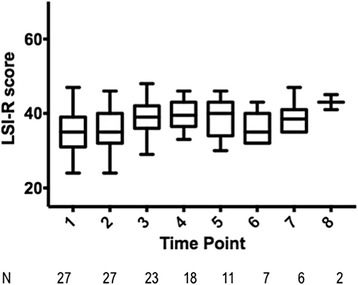
Boxplot of Level of Service Inventory Scores over Time

Analysis of the LSI-R scores over time did not reveal significant trends (Fig. 3). Twenty-seven women completed the LSI-R more than once (*N* = 121 assessments), and trends did not vary significantly beyond the mean. There were no significant correlations in LSI-R scores between contiguous time points, likely related to a lack of variation. Mean intake LSI-R scores did not differ significantly from reassessment (38.4 [SD 5.0] versus 38.9 [SD 9.2], *p* = 0.71, *n* = 22) nor discharge LSI-R scores (36.7 [SD 3.2] versus 37.9 [SD 8.0], *p* = 0.56, *n* = 10). Time on probation was not associated with significant changes in LSI-R scores over time in our study sample population. Italicized values represent statistically significant difference.

## Discussion

In this cross-sectional study of 31 women on probation and living with or at-risk for HIV, we found few significant associations between clinical and CJ measurements of substance use, mental health, social support, relationships/partnerships, or risk. To our knowledge, this is the first evaluation of concurrent validity between the LSI-R and established clinical assessments of depression, substance abuse, and social support in women on probation. That very few correlations were observed suggests that the LSI-R insufficiently reflects women’s overall physical, psychiatric, and emotional health or needs.

Because criminal justice RNA are typically based on actuarial tables, they may misestimate women’s risk and needs with tangible impact on women’s criminal sanctions and supervision case plans (Ostermann & Herrschaft, 2013; Smith et al., 2009). Poor measurement qualities of LSI-R subscales additionally inflate risk by combining fixed and dynamic factors (Via & Dezember, 2016) and do not meet criteria for psychometrically sound scales. The LSI-R is inherently limited by reporting and social desirability biases; clients may perceive far greater repercussions to reporting ongoing substance use, for example, to a probation officer than to a clinician or researcher, even if actual substance use was corroborated by urine toxicology. Unrecognized and untreated substance use and psychiatric disorders are major contributors to poor health and CJ outcomes, and therefore CJ RNA are designed to screen for these substance use and psychiatric disorders. While clinical/research institutions and CJ systems would ideally collaborate to improve individual and public health and safety (Rich et al., 2011), potential cross-talk is limited if goals and measures are not aligned. As a result, women interfacing with both health and CJ systems may have to manage competing institutional priorities, instead of the systems themselves shifting to be more women-centered.

The use of RNA in CJ settings is recommended to identify risk levels as well as service needs. This study illustrates that while RNA are important, there is more work to be done to ensure that the tools serve their multi-dimensional purposes: 1) to identify risk level to ensure that women are not exposed to over-supervision in probation/parole settings; 2) to identify need factors that can be modified to reduce involvement in the CJ system and improve overall functioning; and 3) to tailor services to the unique needs of the individual. Many of the existing RNA tools, like the LSI-R, were developed over 30 years ago and therefore do not reflect this focus on the unique needs of individuals. In contrast, the Women’s Risk Needs Assessment (WRNA), for example, is a more recent tool designed to detect gender-specific needs (e.g., in-depth measures of psychiatric disorders; abuse and trauma histories) and strengths such as self-efficacy (Voorhis et al., 2010). These domains on the WRNA more closely parallel clinical assessments. More individualized and health-focused tools have the power to simultaneously improve people’s health and CJ outcomes, especially if periodically quality-assured, delivered by well-trained staff, and when used (as intended) to inform case plans (Viglione et al., 2015). But this study has shown that more sophisticated RNA tools are additionally needed to ensure that clients receive appropriate services.

Our evaluation of 108 CJ RNA available over 9 years of observation revealed minimal variation in risk scores beyond the mean and no significant trend in scores over time. We observed no difference between initial and reassessment LSI-R scores or initial and discharge scores, contrary to expected declining LSI-R scores over time as a result of targeted case plans (Schlager & Pacheco, 2011; Raynor, 2007). This finding may be due to the quality of the LSI-R administration or it could be due to misidentification and under-treatment of behavioral health needs which generally exacerbated the women’s conditions.

To date, little research has focused on probation recidivism among women with or at-risk for HIV; this work begins to address that gap. Women with 2–4 recurrent probation terms had significantly shorter time to recidivism and higher LSI-R scores than those with fewer probation terms, which is consistent with observations of larger, more diverse cohorts (Vose et al., 2013). Our model adjusted for age, race and number of previous probation periods because these are established risk factors for recidivism in people living with and at-risk for HIV (Baillargeon et al., 2010; White et al., 2008). Older age and more previous probation terms served accounted for a significant increase in the hazard of probation recidivism. Our model indicated that some high school education and no previous inpatient admissions for psychiatric issues were protective against probation recidivism, which aligns with previous studies (Fu et al., 2013; White et al., 2008). Perhaps unexpectedly, we found that a history of prior assault was protective against probation recidivism. Since violent assault is associated with prison/jail recidivism (Fu et al., 2013), this offense may be more likely to result in an incarceration rather than a probation sentence. Further qualitative data is needed to better understand why women perpetrate violence and how this impacts their lived experiences of interacting with CJ systems, applying theory-driven approaches to fully understand people’s behavior.

Despite the novelty of our analytic approach that explored the concurrent validity of criminal justice RNA and clinical assessments using standardized instruments and our inclusion of all RNA assessments over nearly a decade of observation, this study was limited primarily by small sample size. This may have limited our power to detect a significant difference between scores over time, though our findings are consistent with those of larger studies of women. Decreased concurrent validity between the CSSD surveys and clinical assessments could represent social desirability bias since participants may provide more honest responses to clinical researchers as opposed to probation officers. Future studies could examine whether participant responses vary with the position of the interviewer. Generalizability may also be limited by the small sample size. While the demographics of our sample reflects that of women on probation in the state (the population from which the sample was derived), non-Hispanic white and highly education women may be over-represented here compared to other CJ settings. As a proof of concept, however, our work was designed primarily to generate further discussion about the utility of RNA for women with complex health needs and to direct areas for future research. As CJ reform is implemented to decarcerate and keep women in the community longer, it will become ever more important to accurately measure and intervene to address women’s needs.

## Conclusions

In this cross-sectional study of women on probation, widely used RNA tools poorly reflected women’s complex medical, psychiatric, and substance use needs and varied minimally over time, which potentially undermines efforts to direct case plans and reduce recidivism. Future studies should evaluate the ways in which a more nuanced, individualistic approach to evaluating and modifying women’s complex needs can simultaneously improve health and CJ outcomes.
